# Supplementary material for: Decreased bioefficacy of long-lasting insecticidal nets and the resurgence of malaria in Papua New Guinea
Source: Nat Commun. 2020 Jul 20;11:3646. doi: 10.1038/s41467-020-17456-2 (PMC7371689; doi:10.1038/s41467-020-17456-2)
Supplement: Supplementary file 2 — Supplementary Information [file 41467_2020_17456_MOESM2_ESM.pdf]

Supporting Information - Decreased bio efficacy of long-lasting insecticidal nets coincides with resurgence of malaria in Papua New Guinea

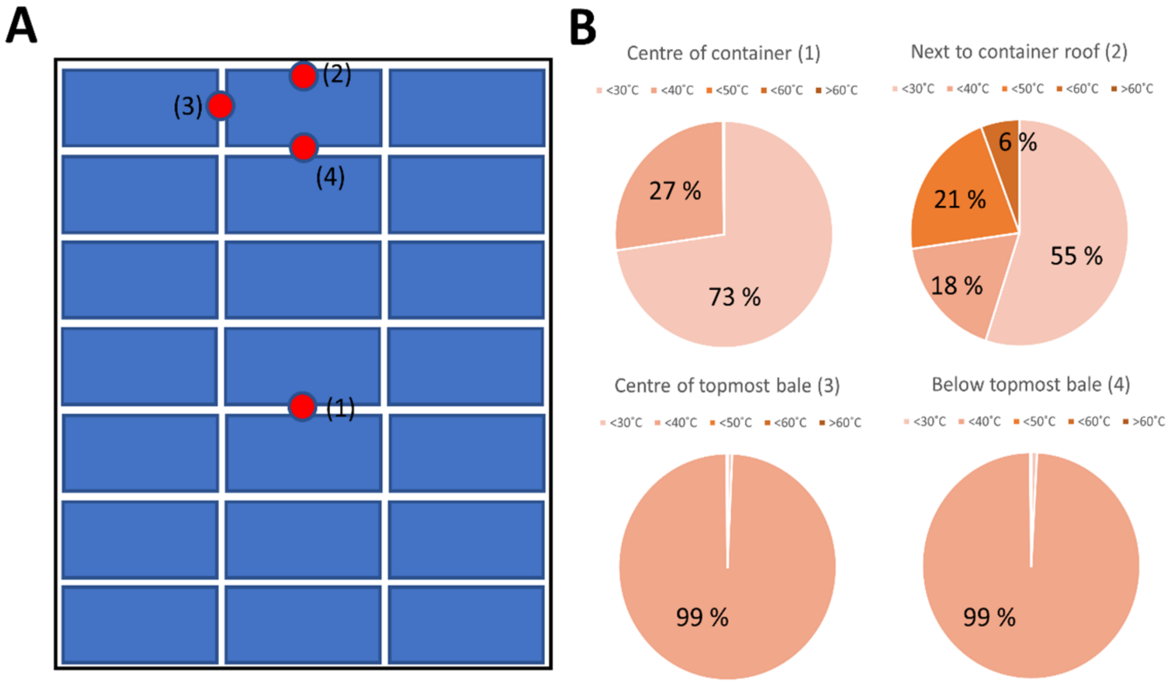

Supplementary Fig. 1: Temperature in LLIN shipping containers in Port Moresby. Temperature was measured for 5 consecutive days (19-23rd Feb. 2019) in 10-minute intervals in 4 locations in the container filled with LLIN bails. Panel A shows an illustration of the front view of a container with the temperature probe locations. Panel B shows the temperature distribution for recorded by each temperature logger. Only below the immediate top ceiling of the container did temperature ever exceed 50 degrees Celsius (6% of the time i.e., approximately an average of 1.5h per day

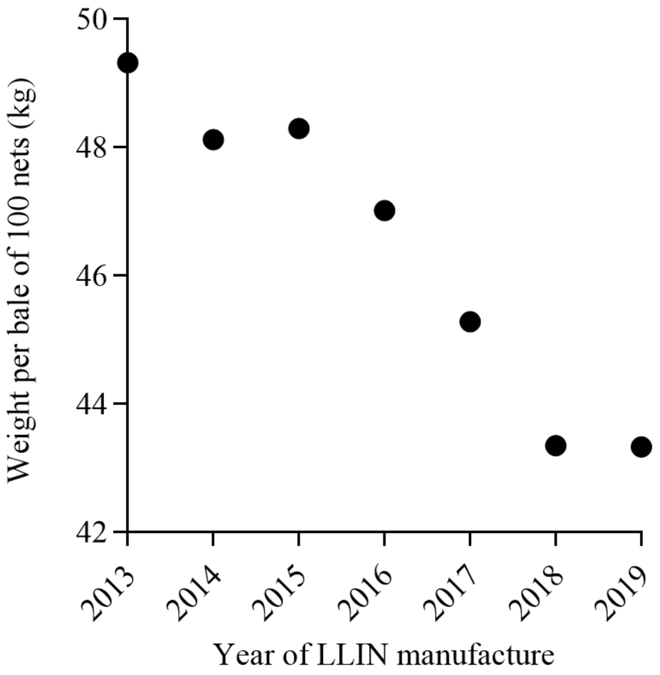

Supplementary Fig. 2: Average weight of LLIN bails decreasing over the years from 2013 to 2019. The overall decrease in weight per LLIN is approximately 70 g.

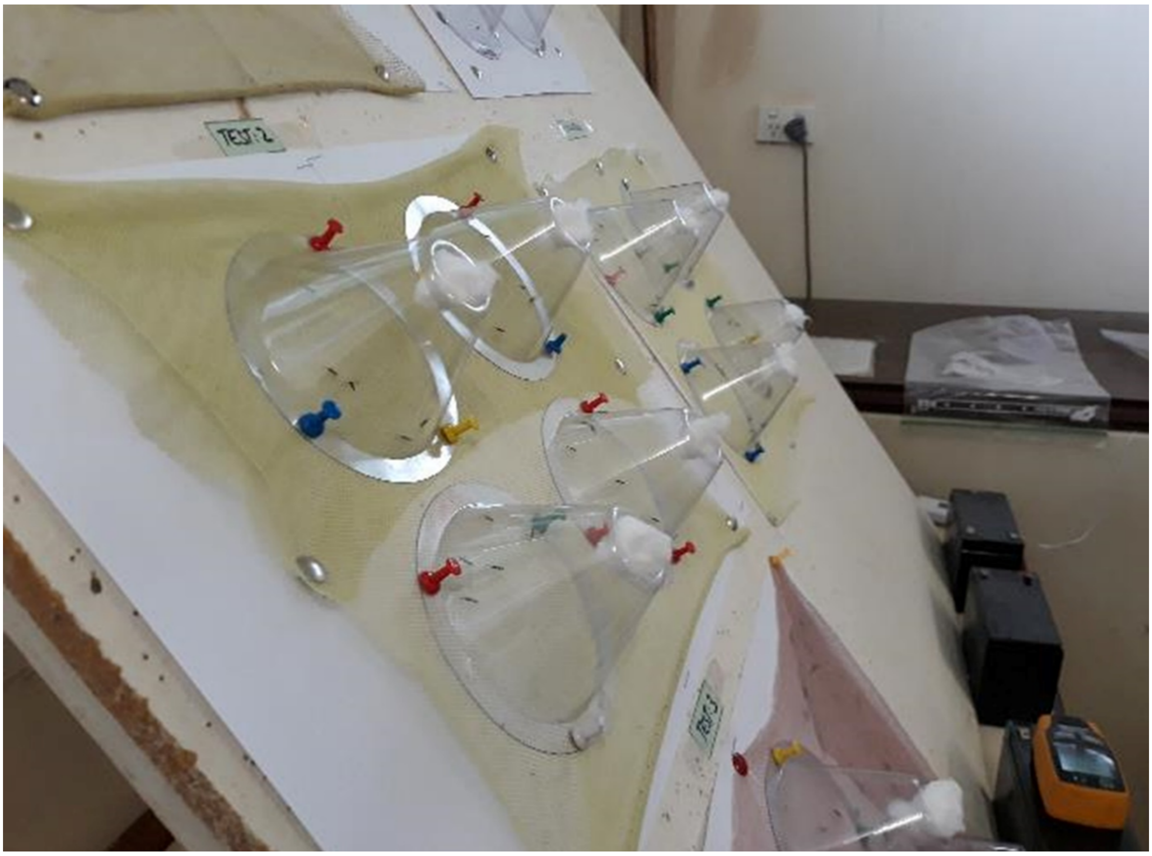

Supplementary Fig. 3: Photograph showing standard WHO cone bioassays being conducted at the PNG Institute of Medical Research Entomology Laboratory in Madang, PNG. The photograph was taken by the authors as part of this study.

Supplementary Table 1: Summary of LLIN collection locations (sourcing provinces or consignment destinations).

Province/Year of manufacture	2007	2008	2009	2010	2012	2013	2014	2015	2016	2017	2018	2019	Total per province
no province specified								2			2		**4**
Central										1			**1**
Chimbu		1		3	4	8	1			19			**36**
New Britain								3				5	**8**
East Sepik					1			1		4	13		**19**
Eastern Highlands	1			2	4	6	3	1	4	29			**50**
Gulf			1		1			10					**12**
Hela											7		**7**
Manus												1	**1**
Morobe							4						**4**
New Ireland		2	1		2			7	4			4	**20**
Oro												6	**6**
Southern Highlands											5		**5**
Western												6	**6**
Western Highlands				2		6	1		3	1			**13**
**Total per year of manufacture**	**1**	**3**	**2**	**7**	**12**	**20**	**9**	**24**	**11**	**54**	**27**	**22**	**192**
