# Supplementary material for: Decreased bioefficacy of long-lasting insecticidal nets and the resurgence of malaria in Papua New Guinea
Source: Nat Commun. 2020 Jul 20;11:3646. doi: 10.1038/s41467-020-17456-2 (PMC7371689; doi:10.1038/s41467-020-17456-2)
Supplement: Supplementary file 3 — Descriptions of Additional Supplementary Files [file 41467_2020_17456_MOESM3_ESM.pdf]

Description of Additional Supplementary Files

Supplementary Data 1

Description: All data collected in the present study are provided in the form of a Microsoft Excel Spreadsheet (Supplementary Data 1.xlsx). The spreadsheet contains one worksheet each for the n=192 unused LLINs and the n=40 LLINs tested in this study, respectively
