# Supplementary material for: Decreased bioefficacy of long-lasting insecticidal nets and the resurgence of malaria in Papua New Guinea
Source: Nat Commun. 2020 Jul 20;11:3646. doi: 10.1038/s41467-020-17456-2 (PMC7371689; doi:10.1038/s41467-020-17456-2)
Supplement: Supplementary file 5 — Reporting Summary [file 41467_2020_17456_MOESM5_ESM.pdf]

nature research

Corresponding author(s): Stephan Karl

Last updated by author(s): Jun 23, 2020

Reporting Summary

Nature Research wishes to improve the reproducibility of the work that we publish. This form provides structure for consistency and transparency in reporting. For further information on Nature Research policies, see our Editorial Policies and the Editorial Policy Checklist.

Statistics

For all statistical analyses, confirm that the following items are present in the figure legend, table legend, main text, or Methods section.

n/a	Confirmed	
☐	☒	The exact sample size (*n*) for each experimental group/condition, given as a discrete number and unit of measurement
☐	☒	A statement on whether measurements were taken from distinct samples or whether the same sample was measured repeatedly
☐	☒	The statistical test(s) used AND whether they are one- or two-sided<br>*Only common tests should be described solely by name; describe more complex techniques in the Methods section.*
☒	☐	A description of all covariates tested
☐	☒	A description of any assumptions or corrections, such as tests of normality and adjustment for multiple comparisons
☐	☒	A full description of the statistical parameters including central tendency (e.g. means) or other basic estimates (e.g. regression coefficient) AND variation (e.g. standard deviation) or associated estimates of uncertainty (e.g. confidence intervals)
☐	☒	For null hypothesis testing, the test statistic (e.g. *F*, *t*, *r*) with confidence intervals, effect sizes, degrees of freedom and *P* value noted<br>*Give P values as exact values whenever suitable.*
☒	☐	For Bayesian analysis, information on the choice of priors and Markov chain Monte Carlo settings
☒	☐	For hierarchical and complex designs, identification of the appropriate level for tests and full reporting of outcomes
☐	☒	Estimates of effect sizes (e.g. Cohen's *d*, Pearson's *r*), indicating how they were calculated

Our web collection on statistics for biologists contains articles on many of the points above.

Software and code

Policy information about availability of computer code

Data collection	Data were collected using the Epicollect 5 (Imperial College, London) open access electronic data capture system.
Data analysis	Data were analysed using Microsoft Excel 2016 (Microsoft Inc.) and GraphPad Prism 8.0 (GraphPad Software). No custom computer code was used.

For manuscripts utilizing custom algorithms or software that are central to the research but not yet described in published literature, software must be made available to editors and reviewers. We strongly encourage code deposition in a community repository (e.g. GitHub). See the Nature Research guidelines for submitting code & software for further information.

Data

Policy information about availability of data

All manuscripts must include a data availability statement. This statement should provide the following information, where applicable:

- Accession codes, unique identifiers, or web links for publicly available datasets
- A list of figures that have associated raw data
- A description of any restrictions on data availability

Source data for all tables and figures are provided with this paper, available as Source Data.xlsx. Figures 1, 3 and 4 contain raw data. All data collected in this study are available in the form of an excel spreadsheet provided as Supplementary Data 1 or under https://doi.org/10.6084/m9.figshare.12552137.v1.

Field-specific reporting

Please select the one below that is the best fit for your research. If you are not sure, read the appropriate sections before making your selection.

- [x] Life sciences
- [ ] Behavioural & social sciences
- [ ] Ecological, evolutionary & environmental sciences

For a reference copy of the document with all sections, see nature.com/documents/nr-reporting-summary-flat.pdf

Life sciences study design

All studies must disclose on these points even when the disclosure is negative.

Sample size	A total of n=192 unused long-lasting insecticidal nets (LLINs) were tested in this study and n=4787 mosquitoes were exposed to these unused LLINs. A total of n=40 used LLINs were also tested in the present study using 811 mosquitoes. Since this was an exploratory study, sample size was not pre-calculated and subject to the availability of LLIN samples. We tested as many LLINs as we were able to source from the wide range of years for which we present data. Statistical analysis presented in the manuscript and post-hoc power calculations show overwhelming significance of observed differences, indicating that our sample size is sufficient.
Data exclusions	Data were excluded only when the mosquito mortality in the negative control exceeded 10%. In this case the bioassay was repeated using a new cutting for the test LLIN and a new untreated net as negative control.
Replication	n=19 LLIN pieces were sent to Liverpool School of Tropical Medicine (LSTM) and re-tested blind. After the tests were completed the net labels were revealed and the results were correlated with the original result - this is shown in Figure 4 in the manuscript. This is an independent validation of the main data. It should be noted that LLINs from 2012 served as positive controls in all assays of the present study. All positive controls consistently showed 100% knock-down and 100% mortality in repeated measurements. For n=5 nets from 2007,2008,2009,2010 and 2012 we tested 5 replicate cuttings per LLIN. All these replications very closely resembled the original data i.e., replications were successful.
Randomization	2019 LLIN samples were randomly selected from either the coolest part of the storage containers or obtained from village communities accross PNG in no particular order. For earlier years we tried to obtain as many nets as possible from village communities. Random side panels were chosen to cut the LLIN sections for bioassay testing.
Blinding	Blinded re-testing of s subset of LLINs was done at LSTM to confirm results.

Reporting for specific materials, systems and methods

We require information from authors about some types of materials, experimental systems and methods used in many studies. Here, indicate whether each material, system or method listed is relevant to your study. If you are not sure if a list item applies to your research, read the appropriate section before selecting a response.

Materials & experimental systems

n/a	Involved in the study	
x		Antibodies
x		Eukaryotic cell lines
x		Palaeontology and archaeology
	x	Animals and other organisms
x		Human research participants
x		Clinical data
x		Dual use research of concern

Methods

n/a	Involved in the study	
x		ChIP-seq
x		Flow cytometry
x		MRI-based neuroimaging

Animals and other organisms

Policy information about studies involving animals; ARRIVE guidelines recommended for reporting animal research

Laboratory animals	no laboratory animals were used in this study.
Wild animals	Wild-caught female Anopheles farauti mosquitoes from Madang Province, PNG were used. Mosquitoes were collected as larvae by sampling larval habitats (dipping),transported and reared to adults in standard mosquito larval containers or adult cages and used in experiments when adults were 2-5 days old. All mosquito samples used in the insecticide bioassays were preserved by storage in ethanol at -20 degrees Celsius for further analyses.
Field-collected samples	Mosquitoes were collected as larvae and reared using standard methods in plastic larval trays and kept in mosquito cages before the experiments. Larvae were fed with ground fish food (Marine Master, Tropical Fish Flakes) and adults were fed 10% sugar solution.
Ethics oversight	No ethics approval is required for work with mosquitoes in PNG or Australia. No human subjects or other animals were involved in this study.

Note that full information on the approval of the study protocol must also be provided in the manuscript.
